# Why Do Individuals with Diabetes Miss Their Dietitian Appointments? A Mixed-Methods Study on Barriers and Strategies for Improved Engagement in Diabetes Care

**DOI:** 10.3390/healthcare13121409

**Published:** 2025-06-12

**Authors:** Lærke P. Lidegaard, Andrea A. Petersen, Bettina Ewers

**Affiliations:** 1 Department of Diabetes Care, Copenhagen University Hospital, Steno Diabetes Center Copenhagen, DK-2730 Herlev, Denmark; bettina.ewers@regionh.dk; 2 Department of Administration, Copenhagen University Hospital, Steno Diabetes Center Copenhagen, DK-2730 Herlev, Denmark; andrea.aaen.petersen.01@regionh.dk; 3 Department of Clinical and Translational Research, Copenhagen University Hospital, Steno Diabetes Center Copenhagen, DK-2730 Herlev, Denmark

**Keywords:** nonattendance, no shows, dietitian appointments, diabetes care, healthcare, chronic disease, engagement, barriers

## Abstract

**Background/Objectives:** Nonattendance at healthcare appointments remains a major challenge, particularly in chronic diseases like diabetes. Dietary therapy is essential in diabetes care, yet nonattendance at dietitian appointments persists. This study aimed to identify key drivers of nonattendance at dietitian appointments, explore prior experiences with dietary counseling, and determine factors motivating attendance. **Methods**: A mixed-methods approach was used in this quality improvement project, drawing on multiple data sources to explore nonattendance at dietitian appointments. This included combining a retrospective analysis of clinical and attendance data from patient records at a Danish outpatient diabetes clinic with semi-structured interviews with 25 individuals who had recently missed a dietitian appointment. Quantitative and qualitative data were analyzed separately and then integrated to characterize overall nonattendance patterns. Interview data were analyzed using systematic text condensation. **Results**: Individuals who missed dietitian appointments were also more likely to miss other healthcare appointments. Vulnerable individuals (i.e., those with complex health conditions or mental health issues) were more likely to miss appointments. Four principal barriers to attendance were identified: administrative, digital, and logistical challenges; competing health concerns; personal priorities; and unclear referral communication and patient involvement. **Conclusions**: Improving attendance at dietitian appointments requires a multifaceted approach. Key recommendations include optimizing scheduling practices, implementing digital reminders, offering continuity of care and virtual consultation options. Referring clinicians should improve patient communication by clearly explaining the purpose of the dietitian consultation and involving patients in shared decision-making prior to referral. Dietitians should collaborate with patients to establish realistic, personalized goals to foster engagement in their diabetes management.

## 1. Introduction

Diabetes is a chronic condition requiring lifelong care. Structured self-management education and support (DSMES) is widely recommended to strengthen self-care behaviors, enhance metabolic control, and improve overall well-being [1]. This approach is dependent on cross-disciplinary collaboration among healthcare professionals to deliver comprehensive person-centered support [1]. The global and national rise in the prevalence of diabetes, particularly among individuals with a lower socioeconomic status, has placed increasing pressure on healthcare systems due to greater demand for medical treatment, management of complications, and long-term care, as well as broader societal costs linked to reduced workforce participation, productivity loss, and increased reliance on social services [2,3,4]. This trend places increasing strain on the healthcare system, necessitating the strategic prioritization of care delivery [5]. The evidence underscores the importance of continuous care and behavioral support to improve treatment outcomes and promote long-term adherence [6,7].

International guidelines advocate for ongoing dietary education and support from a dietitian specialized in diabetes as a key component of DSMES [1,8]. However, unlike routine appointments with diabetes nurses and endocrinologists, dietitian consultations are not offered regularly in Denmark [9]. Instead, referrals to a dietitian are typically made based on the clinical judgment of other healthcare professionals, often triggered by unmet treatment goals, the emergence of complications (medical, physical, or psychosocial), or major transitions in life or care [10,11]. Dietary therapy is recommended to address these challenges, improve clinical outcomes, and equip individuals with the knowledge and skills needed for effective diabetes self-care [1,8]. Dietitian-led dietary therapy has been associated with improved disease management and a reduced need for medication [7,12,13]. Consequently, missed dietitian appointments may negatively affect long-term treatment outcomes and quality of life. Evidence suggests that in individuals with type 1 diabetes (T1D), missed appointments can lead to poorer glycemic control and a higher risk of complications [14,15,16].

However, maintaining consistent engagement with healthcare appointments remains a challenge in diabetes care, both in Denmark and globally [10,11]. Reported nonattendance rates for diabetes-related healthcare appointments range from 4% to 64% [15,17], with nonattendance rates for dietitian appointments reaching up to 36% [14]. The proportion of missed appointments varies according to how nonattendance is defined. In Denmark, up to one-third of all scheduled healthcare visits are missed [18]. This not only affects individual patients but also undermines healthcare system efficiency by contributing to longer wait times, underutilization of clinical resources, and delays in care provision for others [19]. Individuals with chronic conditions such as diabetes are particularly prone to missing outpatient appointments [20]. 

As such, nonattendance has been associated with poorer clinical outcomes, increased strain on healthcare resources and higher costs [15,17,21]. Research suggests that missed appointments in healthcare systems have multifaceted causes. Identified predictors of nonattendance include lower socioeconomic status, younger age, long travel distances, and administrative barriers [22]. In the context of diabetes care, nonattendance has been linked to suboptimal self-care and poorer glycemic control, with contributing factors including younger age, ethnic minority background, poorer health status, and delays in scheduling [15,23]. In diabetes education programs, cultural and emotional factors—such as denial, a lack of perceived need, or negative attitudes toward diabetes education—have also been found to contribute to nonattendance [24]. While these barriers are well-documented, limited research has explored the factors behind missed dietitian appointments, making it challenging to address nonattendance. A qualitative study from 2003 highlighted that patients’ views on the value of consultations, their health locus of control, and a felt obligation to attend dietitian appointments all affected attendance among individuals with diabetes [14]. Other patient-related factors including forgetfulness, low health literacy, and a lack of insight into one’s condition further contribute to missed appointments [25]. These challenges are particularly relevant in dietary care, where consultations often involve sensitive topics and depend on a trusting patient–provider relationship. Moreover, sustaining dietary changes over the long term poses a considerable challenge for individuals with chronic conditions like diabetes due to the emotional burden and the complexity inherent in dietary recommendations [26,27]. Enhancing patient engagement in dietary care requires a deeper understanding of individuals’ past experiences with dietary changes and dietitians, as well as their preferences for future consultations. These insights can inform the development of more personalized dietary care, improved treatment strategies, increased adherence, and, ultimately, clinical improvements.

Thus, addressing nonattendance requires a deeper understanding of the underlying factors, particularly within the context of dietitian services. This study aimed to identify key drivers of nonattendance at dietitian appointments, explore prior experiences with dietary counseling, and determine factors motivating attendance. By addressing these objectives, this study seeks to provide a deeper understanding of barriers to effective engagement in dietary therapy and support the development of strategies to improve attendance and healthcare delivery in diabetes care.

## 2. Materials and Methods

### 2.1. Study Design

This quality improvement project employed a mixed-methods approach, drawing on multiple data sources to gain a comprehensive understanding of nonattendance at dietitian appointments. Quantitative and qualitative data were analyzed separately and integrated to characterize overall nonattendance patterns. 

### 2.2. Data Collection

Data collection involved to separate datasets with data extracted from electronic medical records (EMR) at Steno Diabetes Center Copenhagen (SDCC), a large outpatient diabetes clinic in Denmark’s capital region. One dataset was used for quantitative descriptive analyses, while the other facilitated the identification and recruitment of participants for the qualitative component of the study.

In our study, nonattendance at appointments was defined as missing at least one appointment within a predefined data collection period. Same-day cancelations were not considered nonattendance.

The dataset for the descriptive analyses was used to compare the characteristics of non-attendees at dietitian appointments with those of patients without dietitian appointments at the outpatient diabetes clinic, including their attendance patterns for other healthcare appointments over a one-year period (September 2022–August 2023). The analyzed variables included demographic and clinical factors (last recorded body mass index (BMI) and last measured hemoglobin A1c (HbA_1c_), as well as diabetes-related complications based on diagnosis codes for neuropathy, retinopathy, nephropathy, and cardiovascular disease). Attendance records, including same-day cancelations and missed appointments across various healthcare professions, were also examined.

The second dataset was used to identify individuals who had missed a dietitian appointment within the four months preceding the telephone interviews, aiming to explore underlying reasons for missed appointments. A four-month timeframe was selected to minimize recall bias. The initial data extraction (August–October 2023) identified 70 individuals; however, due to insufficient recruitment, a second extraction (December–February 2024) identified 86 individuals. Telephone interviews were chosen for their flexibility and cost-effectiveness, particularly in reaching individuals who had missed appointments.

Telephone interviews were conducted between December 2023 and May 2024. The research team consisted of four academics with extensive experience in qualitative research, specializing in user involvement, public health, and nutrition. One researcher had professional experience as a dietitian. Additionally, a dietitian student contributed to data collection. To minimize response bias, interviewers introduced themselves solely as researchers, without mentioning their educational backgrounds. None had prior relationships with the participants. Interviews were conducted one-on-one, audio-recorded, and lasted between 11 and 43 min. No repeat interviews were conducted.

#### Interview Guide

The interviews followed a semi-structured interview guide, which included both close-ended questions with fixed answer categories to ensure consistency and uniformity across participants, as well as open-ended questions to allow for more detailed and nuanced responses. Although the interview guide was semi-structured and flexible, all participants were asked the same core questions. Minor deviations and follow-up questions were included in each interview to explore individual responses in greater depth. To ensure consistency, all interviewers received standardized training, observed each other conducting initial interviews, and followed the written procedural guidelines included in the interview guide. This approach is particularly suitable for exploring complex issues in greater depth. To ensure the interview guide covered all relevant aspects, a workshop was conducted in October 2023 with experienced registered dietitians from SDCC who provide dietary therapy for individuals with T1D and type 2 diabetes (T2D). The facilitators, LPL, and an academic colleague with expertise in user involvement and workshop facilitation, guided the session. The dietitians provided valuable insights into why individuals miss their appointments, helping refine the interview guide and identify potential areas for improvement in both the organization and dietitian consultations. The 60 min workshop centered on two key questions: (1) What barriers do you perceive from the patient’s perspective? and (2) What barriers do you perceive related to the organization of dietitian appointments? Facilitators encouraged active participation by using open-ended questions and direct prompts, allowing the dietitians to share their observations and experiences. The discussion was structured using the double diamond model to explore these issues systematically [28,29]. Building on insights from the workshop, the interview guide focused on three primary themes: (1) factors contributing to missed dietitian appointments; (2) prior experiences with dietitian consultations; and (3) patients’ perspectives on virtual consultations. The interview guide was iteratively developed and refined through discussions with the research team prior to its implementation. Definitions and interpretations of the various questions were thoroughly reviewed to ensure a shared understanding among all interviewers.

### 2.3. Recruitment for Interviews

Participants were recruited from the Department of Diabetes Care at SDCC. Eligible participants were Danish-speaking individuals with T1D or T2D who had missed a dietitian appointment at SDCC up to four months preceding the telephone interviews. Individuals with insufficient Danish language skills were excluded. Purposeful sampling was used to ensure a diverse representation of diabetes types, ages, and genders [30]. Interviewers informed potential participants about the study’s objectives, the use of pseudo-anonymity, the voluntary nature of participation, audio recording and data publication. Verbal recorded informed consent was obtained before the interviews began. For participants unable to take part in an immediate telephone interview, a convenient time was scheduled. The sample size was guided by the concept of data saturation, defined as the point at which no new information or themes emerged from the data [31,32].

### 2.4. Interviewed Participants

A total of 138 individuals were contacted for participation. Of these, 87 could not be reached, 9 were excluded due to limited Danish language skills, 1 participant withdrew, and 16 were unable to participate due to scheduling conflicts, e.g., vacation, relocation, work, family illness, or hospitalization. Data collection concluded after interviews with 25 participants, as data saturation was considered reached.

### 2.5. Data Analyses

All telephone interviews were recorded using Puzzel Agent (Puzzel A/S, Oslo, Norway), and note-taking was facilitated using the digital application SurveyXact (Ramboll Management Consulting, Ramboll Group A/S, Copenhagen, Denmark) during the interviews. A data-driven approach guided by systematic text condensation, inspired by Giorgi and modified by Malterud [33], was used for data analysis. Initially, LPL listened to the interviews to gain an overall impression of the data, followed by a detailed review to identify themes by pinpointing statements or meaning units (e.g., words, phrases, or sentences) that described participants’ reasons or explanations for missing their dietitian appointments. The interviews were not transcribed verbatim. LPL maintained a manual interview log to document relevant statements and quotations, while AAP concurrently reviewed the responses and notes collected in SurveyXact. This process helped form an overall impression and independently identified statements and meaning units.

Identified themes were refined in collaboration with BE and the two other academics who had conducted the interviews. Regular discussions between LPL, AAP, and BE ensured that themes were distinct, meaningful, and aligned with study objectives. The final themes were validated against the original recordings, with data saturation reached when themes recurred across interviews. Example quotes were translated into English, lightly edited for clarity, and reviewed by the co-authors for accuracy.

### 2.6. Ethics Considerations

According to the Danish National Committee on Health Research Ethics (reference number F-23058413), qualitative health research of this nature does not require ethical approval from the Regional Ethics Committee of the Capital Region of Denmark [34]. The extraction of data from the EMR was approved locally as the study was classified as a quality improvement project aimed at improving patient attendance at dietitian appointments in the outpatient diabetes clinic. The study adheres to the Consolidated Criteria for Reporting Qualitative Research (COREQ) [35].

## 3. Results

### 3.1. Findings from Descriptive Data

Of the 25 participants interviewed, 68% had T1D, while the remaining participants had T2D. The background characteristics of the participants are summarized in Table 1. Most participants (72%) were male, and one participant chose not to disclose their biological gender. The mean age of the participants was 47 years (range 17–79), with one participant under the age of 18. Six participants had missed their initial dietitian appointment, while nineteen had missed their follow-up appointment. Seven had no recorded diabetes-related comorbidities, whereas three or more with T2D had more than two. Most participants (*n* = 16) with T1D were treated with multiple daily injections (MDIs) of insulin therapy with two participants in the process of transitioning to insulin pump therapy. All participants with T2D were using various types of antidiabetic medication, either alone or in combination.

As shown in Table 2, study participants were slightly older, predominantly male, had more complications, poorer glycemic control, and fewer missed dietitian appointments compared with all of the patients who missed dietitian appointments in the outpatient clinic over the one-year period. Missed or canceled appointments with endocrinologists or diabetes nurses ranged from 0 to 31 visits.

A comparison of patients with missed dietitian appointments and the overall outpatient clinic population with no dietitian appointments (see Supplementary, Table S1) over the one-year period showed that those who missed dietitian appointments were younger, had slightly poorer glycemic control, a marginally higher BMI, and included a higher proportion of individuals with T1D. Their attendance patterns also indicated a greater frequency of missed or canceled appointments with other healthcare professionals.

### 3.2. Qualitative Findings

Four overarching themes were identified as reasons for missed dietician appointments: (1) administrative, digital, and logistical challenges; (2) competing health concerns; (3) other personal priorities in daily life; and (4) unclear referral communication and patient involvement in dietitian care. Additionally, three themes were identified as factors influencing attendance to dietitian appointments: (1) balancing the ideal with the practical possible, (2) past negative experiences and the need for continuity in dietitian care, and (3) scheduling and consultation format preferences. A summary of these themes is provided in Table 3.

#### 3.2.1. Reasons for Missed Dietitian Appointments

**Theme 1: Administrative, digital, and logistical challenges**

A combination of administrative, digital, and logistical challenges was identified as key reasons for missed dietitian appointments. Some were related to individual factors (e.g., digital competencies), while others were linked to healthcare system processes.

The first subtheme, *Challenges in receiving and responding to appointment information*, addresses difficulties participants faced in accessing, understanding, or acting on appointment-related communication. Several participants reported being unaware of their scheduled appointment, while others recalled knowing about it but either forgetting or attempting to cancel—mainly by calling the secretary’s office, with a smaller proportion using the secure digital health app ‘MinSP’ for cancelation.

One factor contributing to appointment-related challenges was the use of ‘e-Boks’, a secure digital mail platform widely used in Denmark for sending healthcare appointment invitations. Some participants reported technical difficulties with this system, such as login issues or trouble accessing messages, which may have prevented them from receiving or reviewing appointment information. Approximately one-third of participants indicated challenges related to digital competencies, affecting their ability to navigate the digital mail system effectively:

*“e-Boks is difficult to use and confusing” (Male, T1D, 46 years, MDI treated, missed a follow-up dietitian appointment)*

Some participants reported not consistently checking their ‘e-Boks’ or receiving notifications at inconvenient times, which led to overlooking or forgetting scheduled appointments:

*“It’s easy to overlook an appointment or forget to check e-Boks—especially if the message comes on without any reminders” (Male, T1D, 67 years, MDI and sensor treated, missed a follow-up dietitian appointment)*

*“There’s too much time between receiving the appointment notification and the actual appointment, so you forget your appointment and the information you were given, especially if it’s, for example, three months away.” (Male, T1D, 30 years, MDI treated, missed an initial dietitian appointment)*

Given the high volume of messages from public authorities, the healthcare system, and others in ‘e-Boks’, some participants may have unintentionally overlooked healthcare appointment notifications. One participant explained that missing an appointment could lead to a cycle of repeated missed appointments:

*“If you miss an appointment, you automatically get a new one in e-Boks, which is scheduled far into the future. It then gets buried among other messages, and you might forget it again.” (Male, 31 years, T1D, MDI treated, missed an initial dietitian appointment)*

Many suggested that digital appointment reminders could help improve appointment adherence:

*“It would be smart to have an SMS reminder both when the appointment is scheduled and another SMS shortly before the appointment” (Nondisclosure of gender, T2D, 23 years, missed a follow-up dietitian appointment)*

Others reported administrative issues with the existing digital system:

*“I received an SMS reminder about an appointment at SDCC, but it came too late—my dietitian appointment had already taken place earlier the same day, so I ended up missing it.” (Male, T2D, 62 years, missed follow-up dietitian appointment)*

The second subtheme, *Transportation and support needs*, addresses how practical barriers related to travel and assistance affected participants’ ability to attend appointments. For example, 44% of participants identified the distance to the outpatient clinic as a barrier. Some lacked access to a driver or personal transportation, while others needed accompaniment but did not have the necessary support.

The third subtheme, *Scheduling difficulties*, relates to the timing of appointments and how well they fit into participants’ daily routines. Overall, 36% reported that the timing of appointments impacted their ability to participate. Participants emphasized the need for a more flexible booking system to improve scheduling and enable the involvement of relatives when necessary. Conflicts with early morning or work-hour appointments were commonly cited as barriers to attendance.

**Theme 2: Competing health concerns**

The first subtheme, *Conflicting medical appointments*, describes how a high volume of healthcare obligations can hinder attendance. Some participants reported having numerous healthcare appointments, which at times led to missed appointments at the outpatient clinic due to the overwhelming volume of commitments. General stress—whether from psychological factors or the burden of managing multiple health conditions—also contributed to forgetfulness and a lack of energy to attend scheduled appointments.

*“I have so many [healthcare] appointments every week, it’s almost unbelievable.” (Male, T1D, 54 years, MDI treated, missed follow-up dietitian appointment)*

The second subtheme, *Mental health challenges*, describes how psychological conditions affected the ability to manage and attend appointments. Participants with mental health conditions, such as post-traumatic stress disorder (PTSD) or bipolar disorder, reported challenges managing appointments due to symptoms like poor sleep, low motivation, or feelings of being overwhelmed:

*“I was struggling mentally at the time [of the missed dietitian appointment]. I have PTSD and I am dealing with the municipality. That’s why I don’t have the energy to attend. Sometimes I sleep poorly at night and end up sleeping into the day.” (Male, 58 years, T2D, missed an initial dietitian appointment).*

*“I’m bipolar and have a very irregular sleep pattern. If I have an early appointment, I try to stay awake all night, but sometimes I still end up falling asleep” (Male, T1D, 29 years, missed dietitian video-follow-up).*

The third subtheme, *Acute illnesses and family health*, captures how temporary illness or caregiving responsibilities influenced attendance. Common illnesses, such as influenza and other infections, were frequently cited as reasons for missed appointments. Additionally, illness in close family members (e.g., children with colds or elderly parents) affected some participants’ ability to attend appointments, either due to concerns about spreading illness or the need to care for family members.

**Theme 3: Other personal priorities in daily life**

The first subtheme, *Psychosocial and financial strains*, highlights how psychosocial and economic factors contribute to difficulties in maintaining appointment attendance. Some participants noted that balancing multiple responsibilities during particularly busy periods made it difficult to keep track of appointments. Competing daily commitments, including work or school obligations, often led to forgetfulness, and missed appointments, especially for those with irregular schedules or demanding jobs:

*“I forgot my appointment because I was busy at work.” (Male, T1D, 54 years, MDI treated, missed follow-up dietitian appointment)*

*“I was called into work on the day I was supposed to go to SDCC, so I didn’t attend.” (Male, 28 years, T1D, MDI treated, missed a follow-up dietitian appointment)*

The second subtheme, *Competing demands from work or education*, describes how specific occupational or educational pressures influence attendance. One participant explained that the timing of their final exams caused a lot of stress and competing priorities, resulting in a missed appointment:

*“I had exams during that period, so I was very busy and had a lot on my mind.” (Male, T1D, 30 years, MDI treated, missed an initial dietitian appointment)*

**Theme 4: Misalignment of expectations and perceived need for dietitian care**

Participants’ awareness of their initial referral varied. A total of 88% reported knowing about their referral to a dietitian, and nearly all of these (92%) recalled being referred by either an endocrinologist or a diabetes nurse. Some participants remembered the purpose of their referral, mentioning reasons such as learning carbohydrate counting, improving blood glucose levels, and/or achieving weight loss. A contributing factor is reflected in the first subtheme, *Lack of clarity in interprofessional communication*, which highlights how poorly communicated referral information can undermine patients’ understanding of the role and purpose of dietitian care. However, when reflecting on their interactions with the referring healthcare professional—particularly how the referral was communicated and whether they were involved in the decision—most participants could not recall specific details. Additionally, some reported a lack of information about why they had been referred to a dietitian and expressed uncertainty about the need for dietary counseling. This lack of clear communication contributed to their nonattendance:

The second subtheme, *Perceived relevance of dietitian support*, underlines how uncertainty about the necessity of dietary counseling reduces motivation to attend.

*“I didn’t attend because I didn’t understand the purpose (Male, T2D, 79 years, missed an initial dietitian appointment).”*

*“The purpose wasn’t clear; I think people stay away if they don’t know what they’ll get out of it.” (Female, T1D, 67 years, MDI treated, missed an initial dietitian appointment)*

One participant also expressed a lack of motivation to engage with the dietary methods for which she had been referred and disagreed with the proposed change to her treatment plan:

*“I’m not interested in getting a pump or learning to count carbohydrates.” (Female, T1D, 67 years, MDI treated, missed an initial dietitian appointment)*

Some participants perceived the referral to a dietitian as unnecessary, which lowered their motivation to attend appointments. For others, conversations with other healthcare professionals reinforced their belief that dietary consultations were not relevant at the time, further influencing their decision to miss appointments:

*“I spoke with the nurse—or was it the doctor?—about whether it was relevant for me right now. I felt confident in managing the diet, so I didn’t attend.” (Female, T1D, 17 years, treated with insulin pump treated T1, missed a follow-up dietitian appointment)*

The third subtheme, *Clarity of purpose and perceived value of dietitian support*, reflects how insufficient engagement and unclear goals at the dietitian can negatively influence attendance. Among participants who had missed follow-up appointments, challenges included unclear communication—such as limited information about the purpose of the consultation, vague behavioral goal setting, and inadequate discussion of expected benefits or realistic timelines for improvement. When the purpose of dietary counseling was not clearly reinforced or discussed during sessions, some participants struggled to see its relevance, did not perceive it as being aligned with their individual needs, or felt less motivated to continue when they did not experience quick, noticeable health improvements:

*“I didn’t really feel like I needed nutritional counseling… I don’t know why I was supposed to see a dietitian. Something about my blood sugar, I’m not sure.” (Male, T1D, 19 years, MDI treated, missed a follow-up dietitian appointment)*

*“It was because I didn’t feel like I was getting anything out of it that I missed the follow-up appointment.” (Male, T2D, 68 years, missed a telephone follow-up dietitian appointment)*

#### 3.2.2. Factors Influencing Dietitian Attendance

**Theme 1: Balancing the ideal with the practical possible**

Several participants described a tension between ideal dietary recommendations and the practical realities of implementing them. While many aspired to follow dietary recommendations, real-world limitations such as time, finances, and daily routines made it difficult to maintain. A contributing factor is reflected in the first subtheme, *Gap between recommendations and reality*, which highlights the challenges faced when ideal advice conflicts with everyday life constraints. Some found major dietary changes overwhelming, whereas smaller, more manageable adjustments were perceived as easier to incorporate:

*“It’s not always possible to make the changes the dietitian wants you to make. Unfortunately, not all of us have the finances to eat as healthily as we’d like. But there are still some easier things, like replacing soda or juice with sugar-free options, which were relatively easy to do and didn’t cost more.” (Nondisclosure of gender, T2D, 23 years, missed a follow-up dietitian appointment)*

Some participants described a gap between their taste preferences and the dietary recommendations provided by dietitians. Additionally, some participants felt that the expectations set by dietitians were too high, making it difficult to fully implement the suggested dietary changes:

*“It’s really good [to talk to a dietitian], but I can’t always follow through. Taste also plays a big role—you don’t always choose the healthiest option. I think the bar is set a bit too high”. (Male, T1D, 54 years, MDI treated, missed a follow-up dietitian appointment)*

*“I know what I should do, but I find it hard to make it work in my daily life.” (Female, T2D, 60 years, missed a follow-up dietitian appointment)*

The second and third subthemes, *Maintaining dietary changes* (e.g., *carbohydrate counting*) and *Need for flexible, personalized guidance*, together capture the challenges participants faced in sustaining complex dietary strategies and the value they placed on individualized support.

For many, consistently applying carbohydrate counting in daily life was perceived as time consuming, complex, and demanding ongoing precision and effort. Participants with T1D described how balancing this task alongside work and other responsibilities made it difficult to maintain. In particular, accurately calculating carbohydrate content was challenging in everyday situations, such as eating out, preparing homemade meals, or navigating foods without labels. Tasks like weighing ingredients and researching nutritional information added to the burden, often making the process feel overwhelming:

*“It’s way too complicated [to count carbohydrates] in everyday life, to spend so much time counting. You go to work, you need lunch—it’s just too much trouble. Sometimes you eat out, and it’s way too difficult to count and figure out what contains carbohydrates (Male, T1D, 46 years, MDI treated missed an initial dietitian appointment).*

*“The dietitians provided a good “starter pack”, but from there it gets harder when you have to weigh all your food to count [carbohydrates] and figure out whether they’re fast or slow carbohydrates. You catch yourself weighing your food all the time.” (Male, T1D, 31 years, MDI treated, missed an initial dietitian appointment)*

*“I can’t find the energy to count [carbohydrates] with my work. I can’t get into a rhythm with counting. Inputting data into an app or doing food records—I just completely shut down on that.” (Male, T1D, 28 years, MDI treated, missed a follow-up dietitian appointment)*

When carbohydrate counting felt burdensome or failed to produce immediate, noticeable results, motivation to continue often declined. Some participants also found existing tools impractical or outdated:

*“I can only find the energy to log what I’ve eaten [in a bolus calculator app], but not the insulin I’ve taken, so it ends up being pointless.” (* *Male, T1D, 28 years, MDI treated, missed an initial dietitian appointment)*

*“The techniques [for carbohydrate counting] are a bit outdated, and the app design is old-fashioned. It would be great to have an app that could use the phone’s camera—like, you’re eating a portion of oatmeal, this many carbs—this much insulin you need to take.” (Male, T1D, 31 years, MDI treated, missed an initial dietitian appointment)*

These experiences underscore the *need for flexible, personalized guidance* to support long-term engagement. While some participants found carbohydrate counting challenging, others appreciated how dietitian consultations provided tailored strategies that simplified insulin dosing and helped translate general advice into meaningful, everyday actions:

*“Every time I talk to a dietitian, I learn more about how much insulin I should take for my meals… It’s the math of it that the dietitian helps with.” (Male, T1D, 56 years, MDI treated, missed a virtual follow-up dietitian appointment)*

**Theme 2: Past negative experiences and the need for continuity in dietitian care**

The first subtheme, *Negative past experiences with dietitians or diets*, describes how prior dissatisfaction or negative experiences can impact future attendance. Most participants (84%) had prior experience with dietitian consultations in various settings, including hospitals, primary care, or private practice. Several described negative experiences that impacted their willingness to engage in further appointments with a dietitian:

*“I’m a bit scared. I went to XX [another hospital in Denmark’s capital region] before and was given a 1400 kcal meal plan because I needed to lose weight. I was hungry all the time. I don’t want to go through that again.” (Male, T1D, 30 years, MDI treated, missed an initial dietitian appointment)*

This restrictive plan led to persistent hunger and resistance toward dietary guidance, as it was perceived as unsustainable and dissatisfying.

Other participants recalled experiences with highly restrictive diets or a critical focus on food choices, which emphasized mistakes over progress. These interactions often contributed to feelings of guilt and a strained relationship with food:

*“I had BED [binge eating disorder] as a child, before it was recognized as a diagnosis, and I was put on a lot of very strict diets. I wasn’t allowed to participate in class when there was cake. That has made my relationship with food difficult.” (Gender nondisclosure, T2D, 23 years, missed a dietitian follow-up appointment)*

A few participants also reflected on past experiences of weight-centered messaging. However, one participant acknowledged that this emphasis had shifted in more recent consultations:

*“In the past, you were constantly told that you were fat. The focus was always on what you shouldn’t do. It’s different today.” (Male, T1D, 46 years, MDI treated, missed a follow-up dietitian appointment)*

The second subtheme, *Desire for continuity (seeing the same dietitian)*, underscores the role of stable therapeutic relationships in fostering adherence and enhancing patient comfort. Many participants reported seeing different dietitians at each visit and several emphasized the importance of continuity in dietitian care. They highlighted that establishing continuity in the relationship fostered trust and reduced the need to repeat information at every visit:

*“It would be really nice to have the same dietitian again. I’ve seen two to three different ones, and they’ve asked about the same things.” (Female, T1D, 17 years, MDI treated, missed a follow-up dietitian appointment)*

*“It would be nice to talk to the same person each time, and it would make follow-up better.” (Male, T2D, 65 years, missed a follow-up dietitian appointment)*

**Theme 3: Scheduling and consultation format preferences**

The first subtheme, *Need for flexible scheduling*, captures the participants’ desire for appointment times that better align with their daily routines. One-third of participants reported that they would have been less likely to miss their appointment if it had been conducted via telephone or video consultation. Participants highlighted the flexibility of virtual formats, which allowed them to better manage work and other responsibilities:

*“I could talk to the dietitian during work hours for 15 minutes; I wouldn’t need to take time off and spend nearly two hours on it.” (Male, T1D, 54 years, MDI treated, missed a follow-up dietitian appointment)*

*“I could save transport time, making it more flexible.” (Male, T1D, 31 years, MDI treated, missed an initial dietitian appointment)*

Nearly half of the participants also highlighted that virtual consultations helped avoid the logistical burden of coordinating multiple healthcare appointments at the outpatient clinic:

*“My appointments don’t need to be combined; I can just have a single phone call with the dietitian” (Male, T1D, 29 years, missed video-follow-up).*

Preferences regarding appointment scheduling varied: about half of the participants preferred coordinated, same-day appointments, while the other half favored having their appointments spaced out. These differences suggest that mismatches in scheduling preferences may contribute to missed appointments.

The second subtheme, *Preferences and challenges of virtual consultations*, reflects participants’ mixed experiences with remote appointments. Virtual consultations were generally viewed as a way to enable more frequent follow-up with dietitians. Just under a quarter of participants felt the intervals between appointments were too long, which made it harder to maintain focus on the dietary changes they had committed to. This concern was slightly more common among participants with T2D, while those with T1D expressed a need for more frequent contact during the initial phase of learning carbohydrate counting. After this learning phase, regular appointments were often seen as less essential, though many still appreciated having the option of brief virtual check-ins when needed. This finding aligns with participants’ suggestions for a more user-friendly and flexible booking system.

However, some participants noted that the lack of non-verbal cues in virtual consultations could affect the quality of the interaction. Concerns were also raised about the reduced sense of personal connection:

*“There’s no real dialogue. It’s hard to have a proper dialogue [during telephone consultations]. Body language is important, and you can’t have that in a phone call. It’s also hard for the dietitian to assess if I’m overweight when I say I’m too fat.” (Male, T1D, 44 years, MDI treated, missed a follow-up dietitian appointment)*

*“You will miss the personal connection; the conversation becomes more meaningful when you’re sitting together.” (Male, T1D, 46 years, MDI treated, missed follow-up dietitian appointment).*

## 4. Discussion

This study examined the characteristics and key drivers of nonattendance at dietitian appointments, identifying several factors that could improve engagement and attendance rates, particularly in an outpatient diabetes clinic setting. Descriptive analyses showed that individuals who missed dietitian appointments also tended to miss other healthcare appointments more frequently. The high frequency of missed or canceled visits with diabetes nurses and endocrinologists suggests a high tolerance for rebooking at the outpatient clinic study site among individuals with frequent no-shows and cancelations. Interviews with participants who had missed dietitian appointments revealed several key barriers to attendance, including administrative and logistical challenges, limited digital competencies, competing health concerns, and other daily life priorities. While some participants recalled understanding the purpose of their referral to a dietitian, many pointed to unclear communication—regarding the need for referral and a lack of involvement in this decision-making process—as factors influencing their nonattendance. Additionally, insufficient information reinforcing the purpose of the referral, aligning expectations, and involving patients in goal setting during dietitian consultations was identified as a key factor contributing to nonattendance at follow-up appointments. Critical factors supporting engagement and adherence to dietitian appointments included flexible scheduling, the availability of virtual consultations, continuity in dietitian care, and realistic, personalized dietary guidance.

Participants’ understanding and assessment of the need for dietary therapy played an important role in their decision to attend dietitian appointments. While some perceived dietary changes as essential for managing their health, others did not prioritize them. The literature suggests that a common cause of nonattendance is the failure to perceive certain healthcare appointments as necessary or relevant to immediate needs [36]. A study on dietitians’ experiences found that many individuals are unaware of the importance of dietary changes in diabetes management, leading to low motivation for dietary counseling [37]. Additionally, our findings suggest that doubts about the usefulness of dietary guidance can further decrease engagement. Some of our study participants reported that the purpose of their referral to a dietitian had not been clearly explained, emphasizing the need for more effective communication from healthcare professionals. Pregnant women with diabetes have also been found to face challenges attending dietitian appointments due to unclear communication regarding the purpose of their referral [38]. Similarly, a Danish systematic review [39] identified poor communication as a major factor contributing to nonadherence, stressing the need for clear, concise, and personalized information. By providing concise, individualized explanations of referrals, healthcare professionals can enhance motivation and involvement. Discussing individual expectations—such as motivations for change, willingness to engage with a dietitian, and past negative experiences with dietitians or restrictive diets—should take place prior to referral to help reduce nonattendance. According to the Health Belief Model, an individual’s perception of the benefits of an intervention directly impacts their willingness to engage, making clear explanations of the dietitian’s role crucial for reducing nonadherence rates [40].

Several participants expressed frustration and feeling overwhelmed when attempting to make dietary changes. Although they knew what they should do, they struggled to translate recommendations into concrete actions in daily life. Knowledge alone does not necessarily lead to lasting changes, as habits and routines play an important role. At the same time, dietitians face the challenge of quickly understanding individual needs and preferences while balancing realistic goals with dietary recommendations. If dietary guidance is perceived as unattainable, it can lead to discouragement and an ‘all-or-nothing’ mindset, where individuals either follow the recommendations strictly or give up entirely. This perception can create feelings of failure and stigmatization, which in some cases leads to missed appointments [41]. A needs-based and personalized approach is therefore crucial for maintaining patient motivation. Additionally, participants struggled to reconcile dietitian recommendations with constraints related to finances, time, and taste, frequently finding the expectations unrealistic. Many also found it challenging to implement dietary recommendations, as traditional dietary guidance is often provided in settings that lack support for developing practical food and diabetes management skills. Moreover, eating behaviors are shaped not only by personal choices but also by external factors such as social contexts and marketing [42]. This highlights the need for practice-oriented approaches that are actionable and relevant to daily life, fostering both learning and motivation. These approaches should account for broader structural influences on food decisions and be personalized to individual circumstances to promote sustained engagement and meaningful behavioral change.

Consultations with a dietitian typically combine more technical guidance, such as carbohydrate counting (especially for individuals with T1D), with therapeutic discussions addressing body weight, eating habits, and the emotional and psychosocial factors influencing them (especially for individuals with T2D). While carbohydrate counting is important for optimizing glycemic control, many of the participants struggled to stay engaged with these technical aspects, finding carbohydrate counting overwhelming and difficult. Although closed-loop insulin pump systems have been shown to significantly improve glycemic control, research indicates that accurate carbohydrate estimation remains crucial for further optimizing glucose management [43]. Looking ahead, innovations such as AI-assisted food image recognition for estimating carbohydrate content in meals may offer additional support for individuals with diabetes. However, dietitians will continue to play a critical role in assessing individual needs and tailoring carbohydrate counting strategies, considering factors such as literacy, numeracy, and daily routines. For those who find carbohydrate counting particularly challenging, alternative strategies may be required to achieve better glycemic control, and they may benefit from dietary approaches beyond fixed ratios or precise carbohydrate counting.

Therapeutic dietary consultations can help address the emotional and psychological factors influencing dietary habits, body weight, and past negative experiences with restrictive diets. Changing long-standing behaviors requires a trusting environment where these factors are acknowledged. The relationship with the dietitian and trust in their guidance are key in an individual’s decision to attend appointments [44]. Several participants expressed dissatisfaction with seeing different dietitians at each visit. Research emphasizes the importance of continuity in care [45], suggesting that allowing individuals to wait for their regular dietitian could foster trust, improve follow-up and potentially also dietary adherence. Furthermore, the therapeutic relationship between the dietitian and patient, along with the counseling approach, is fundamental to adherence to the dietary therapy, underscoring the need for personalized care [46].

Several factors in our study identified as causes of nonattendance at dietitian appointments appear to be universal rather than specific to this type of appointment. Practical barriers—such as transportation issues, financial constraints, and social obligations (including work or caregiving responsibilities)—along with physical and mental exhaustion— are common challenges that often lead to missed appointments across various healthcare settings [18,36,47]. These factors suggest that nonattendance is frequently influenced by social conditions, personal difficulties, and previous experiences with the healthcare system. They underscore the multifaceted nature of nonattendance and the need for a comprehensive approach to better understand and address its causes in healthcare settings.

We found that nearly half of the participants were unaware of their missed appointment, with some simply overlooking their digital invitations. This aligns with previous research suggesting that nonattendance is not only a patient-driven issue but also a structural challenge within the healthcare system. For example, one study found that 54% of individuals classified as non-attendees believed the hospital had made an error [48]. Another study found that 44% of non-attendees were unaware of their appointment and claimed they had not received any notification [49]. These findings indicate that nonattendance warrant a closer examination of administrative processes and communication flow to identify potential system errors and gaps contributing to missed appointments. Furthermore, this highlights the need for more comprehensive categories for recording missed appointments and same-day cancelations in electronic health records. Current classifications primarily include hospitalization, death, or the generic “no-show” designation, limiting the ability to analyze the underlying causes of nonattendance. Some participants suggested SMS reminders and more flexible booking systems as strategies to reduce no-shows and improve appointment scheduling. A Cochrane review has shown that SMS messages and self-booking outpatient clinics significantly reduce nonattendance rates [50,51,52]. Despite these promising interventions, machine learning and AI have yet to fully address this issue [53]. However, as AI technology advances, it may help reduce nonattendance by predicting this and enabling targeted reminders or adaptive scheduling.

Virtual consultations are rapidly gaining traction and are increasingly viewed as a necessary advancement in the Danish healthcare sector to enhance accessibility, efficiency, and meet future needs [54]. They offer benefits such as greater flexibility and reduced travel time, yet challenges remain, including technological limitations and a reduction in personal interaction [55,56,57]. Allowing individuals to choose between in-person and virtual appointments has been shown to significantly reduce nonattendance rates [58]. Additionally, combining clear communication about the purpose of the appointment with SMS reminders has been found to greatly improve attendance rates [59]. These findings highlight the importance of flexibility and clear communication in reducing missed consultations. However, as our study found, patients—particularly those with limited digital competencies—need better support and preparation.

Our study provides valuable insights into patients’ perspectives on reasons for missing dietitian appointments. A key strength lies in the inclusion of a diverse group of participants, comprising individuals who missed their initial referral appointment as well as those who missed follow-up appointments, and including both people with T1D and T2D. This diversity enhances the transferability of the findings and supports a more comprehensive understanding of the factors contributing to nonattendance at dietitian appointments during diabetes care. However, despite efforts to recruit a representative sample of the population, we excluded non-Danish speaking patients, a group known to have higher nonadherence rates due language barriers and limited access to digital healthcare services [60]. In addition, the interview sample included more males than females; however, it is well established that men are generally more likely than women to miss their health appointments [25,61]. Another limitation of the study was the reliance on telephone interviews, which restricted access to non-verbal cues and may have introduced selection bias. While all interviews were guided by a semi-structured interview guide, some flexibility was allowed to explore participants’ responses in greater depth. This approach supported both consistency and richness in the data, though it may have led to variation in how topics were covered across interviews. Additionally, while data from participants with both T1D and T2D were included, our findings primarily reflect the experiences of individuals with T1D, who may consult dietitians for fine-tuning of self-care behaviors such as insulin dose adjustments at meals. In contrast, individuals with T2D often require more basic dietary guidance, such as sustained motivation, and tailored support for managing comorbidities or participating in group-based interventions. As such, preferences regarding appointment format, timing, frequency, and content identified in this study may not be directly transferable to the T2D population. Future strategies to reduce nonattendance may benefit from differentiating between types of nonattendances—such as frequent same-day cancelations, occasional missed appointments (partial nonattendance), and complete disengagement from follow-up care (treatment dropout).

Finally, data collection and analysis were partly conducted by healthcare professionals involved in dietary therapy for diabetes, whose clinical perspectives may have influenced both participant interactions and interpretation of findings. To address this, we employed a reflective and transparent approach throughout the research process, including regular discussions with other health researchers and critical review by a co-author without a clinical background to help ensure analytical rigor and minimize potential bias.

## 5. Conclusions

In conclusion, this study identified several factors contributing to missed dietitian appointments among individuals with diabetes offering actionable recommendations for improving attendance. Our findings emphasize the need for a multifaceted approach, addressing both healthcare system and interpersonal factors. From a system perspective, enhancing administrative processes offering digital reminders, flexible scheduling, continuity in dietitian care and virtual consultations can improve accessibility and better accommodate diverse patient preferences. Equally important, effective interpersonal care begins with healthcare professionals providing clear communication and actively involving patients, particularly before referring them to a dietitian. This involves explaining the purpose of dietitian consultations, emphasizing the role of dietary guidance in diabetes management, and addressing any previous negative experiences with dietitians or dietary changes. Dietitians should then build on this foundation by setting realistic goals and expectations to enhance patient motivation and engagement in their diabetes care

## Figures and Tables

**Table 1:** Characteristics of the 25 participants interviewed based on diabetes type.

Type 1 Diabetes (*n* = 17)	Number (%) or Median (IQR, Range)
Biological gender, *n* (%) male	13 (77)
Age, years	42 (IQR: 30–55, range 17–67)
Living with a partner, *n* (%)	6 (36)
Employed, *n* (%)	11 (65)
BMI, m/kg^2^	26 (IQR: 21–32, range 15–42)
Duration of diabetes, years	15 (IQR: 5–23, range 3–29)
HbA_1c_, mmol/mol	70 (IQR: 61–78, range 47–114)
HbA_1c_, %	8.6 (IQR: 7.7–9.3, range 6.5–12.6)
Comorbidities, *n* (%)	11 (65)
Insulin pump	1 (6)
Type 2 Diabetes (*n* = 8)	Number (%) or Median (IQR, Range)
Biological gender, *n* (%) male	5 (63)
Age, years	59 (IQR: 58–66, range 23–79)
Living with a partner, *n* (%)	4 (50)
Employed, *n* (%)	2 (25)1
BMI, m/kg^2^	37 (IQR: 33–41, range 27–43)
Duration of diabetes, years	13 (IQR: 8–16, range 4–28)
HbA_1c_, mmol/mol	70 (IQR: 62–76, range 43–113)
HbA_1c_, %	8.6 (IQR: 7.8–9.1, range 6.1–12.5)
Comorbidities, *n* (%)	7 (88)
Antihyperglycaemics, *n* (%)	8 (100)

Data are presented as medians with interquartile ranges (IQRs; 25th and 75th percentiles) and range. Categorical data are summarized as numbers and percentages. Abbreviations: BMI, body mass index; HbA_1c_, hemoglobin A_1c_; IQR, interquartile range.

**Table 2:** Comparison of participants and all patients missing dietitian appointments over one year.

	Interviewed Participants *n* = 25	All Patients with Missed Dietitian Appointments ^1^ *n* = 528
Biological gender, *n* (%) male	18 (72)	309 (59)
Age, years	50 (IQR: 33–62)	40 (IQR: 29–56)
Diabetes type, *n* (%)		
Type 1 diabetes	14 (64)	358 (68)
Type 2 diabetes	8 (36)	170 (32)
Complications ^2^, *n* (%)		
None	7 (28)	224 (42)
1 complication	10 (40)	186 (35)
2 complications	5 (20)	100 (19)
≥3 complications	3 (12)	18 (3)
Metabolic data		
HbA_1c_, mmol/mol	67 (IQR: 61–77)	62 (IQR: 54–73)
HbA_1c_, %	8.3 (IQR: 7.7–9.2)	7.8 (IQR: 7.1–8.8)
BMI, kg/m^2^	27 (IQR: 22–36)	27 (IQR: 24–31)
Same-day cancellations ≥ 1 time ^3^, *n* (%)	13 (59)	420 (80)
Missed appointments		
All types ^3^, median *n* of missed visits	2.5 (IQR: 1–3)	2.0 (IQR: 1–4)
Dietitian, *n* of missed visits	1.0 (IQR: 1–2), range 1–3	1.0 (IQR:1–2), range 1–5
Endocrinologist, *n* (%)	7 (32)	188 (36)
Diabetes nurse, *n* (%)	2 (9)	177 (34)
Ophthalmic nurse, *n* (%)	5 (23)	92 (17)
Podiatrist, *n* (%)	1 (5)	45 (9)

Data are presented as medians with interquartile ranges (IQRs; 25th and 75th percentiles). Categorical data are summarized as numbers and percentages. Abbreviations: BMI, body mass index; HbA_1c_, hemoglobin A_1c_; IQR, interquartile range. ^1^ Data collected from September 2022 to August 2023. ^2^ Diabetes-related complications; neuropathy, retinopathy, nephropathy, cardiovascular disease. ^3^ All types of appointments incl. dietitians, endocrinologists, nurses, and podiatrists.

**Table 3:** Categories, key themes, and subthemes.

Category	Theme	Subtheme
Reasons for missed dietitian appointments	1. Administrative, digital, and logistical challenges	Challenges in receiving and responding to appointment information; Transportation and support needs; Scheduling difficulties.
	2. Competing health concerns	Conflicting medical appointments; Mental health challenges; Acute illnesses and family health.
	3. Other personal priorities in daily life	Psychosocial and financial strain; Competing demands from work or education.
	4. Misalignment of expectations and perceived need for dietitian care	Lack of clarity in interprofessional communication; Perceived relevance of dietitian support; Clarity of purpose and perceived value of dietitian support.
Factors influencing dietitian attendance	1. Balancing the ideal with the practical possible	Gap between recommendations and reality; Maintaining dietary changes (e.g., carbohydrate counting); Need for flexible, personalized guidance.
	2. Past negative experiences and the need for continuity in dietitian care	Negative past experiences with dietitians or diets; Desire for continuity (seeing the same dietitian).
	3. Scheduling and consultation format preferences	Need for flexible scheduling; Preferences and challenges of virtual consultations.

## Data Availability

The datasets presented in this article are not readily available to preserve anonymity of participants.

## Supplementary Materials

The following supporting information can be downloaded at: https://www.mdpi.com/article/10.3390/healthcare13121409/s1, Table S1: Comparison of dietitian non-attendees and diabetes patients without dietitian visits over one year.

## Abbreviations

The following abbreviations are used in this manuscript:

AAP	Andrea Aaen Petersen
BE	Bettina Ewers
BMI	Body mass index
COREQ	Consolidated Criteria for Reporting Qualitative Research
DSMES	Diabetes self-management education and support
EMR	Electronic medical records
HbA1c	Hemoglobin A1c
IQR	Interquartile range
LPL	Lærke Pinstrup Lidegaard
MDI	Multiple daily injections
SDCC	Steno Diabetes Center Copenhagen
T1D	Type 1 diabetes
T2D	Type 2 diabetes
