# Supplementary material for: Why Do Individuals with Diabetes Miss Their Dietitian Appointments? A Mixed-Methods Study on Barriers and Strategies for Improved Engagement in Diabetes Care
Source: Healthcare (Basel). 2025 Jun 12;13(12):1409. doi: 10.3390/healthcare13121409 (PMC12192737; doi:10.3390/healthcare13121409)
Supplement: Supplementary file 1 [file healthcare-13-01409-s001.zip › Table S1. Supplementary.pdf]

Table S1. Comparison of dietitian non-attendees and diabetes patients without dietitian visits over one year

	Total dietitian non-attendees[1] *n* = 544	Patients with no dietitian visits in the outpatient diabetes clinic[1] *n* = 9,405
Biological gender, *n* (%) male	319 (59)	5,382 (57)
Age, years	40 (IQR: 29-57)	57 (IQR: 34-70)
Diabetes type, *n* (%)		
Type 1 diabetes	367 (67)	5,780 (61)
Type 2 diabetes	177 (33)	3,625 (39)
Complications[2], *n* (%)		
None	229 (42)	3250 (35)
1 complication	192 (35)	3052 (32)
2 complications	104 (19)	2102 (22)
≥3 complications	19 (4)	1001 (11)
Metabolic data		
HbA1c, mmol/mol	62 (IQR: 54-74)	56 (IQR: 49-65)
BMI, kg/m^2	27 (IQR: 24-31)	26 (IQR: 23-30)
Same-day cancellations ≥1 time, *n* (%)[3]	429 (79)	2,489 (26)
Missed appointments		
All types, median *n* of missed visits[3]	2 (IQR: 2-4)	0 (IQR: 0-1)
Dietitian, *n* of missed visits	1 (IQR: 1-2)	–
Endocrinologist, *n* (%)	193 (35)	1368 (15)
Diabetes nurse, *n* (%)	179 (33)	1088 (12)
Ophthalmic nurse, *n* (%)	95 (18)	848 (9)
Podiatrist, *n* (%)	45 (8)	376 (4)

Data are presented as medians with interquartile ranges (IQR; 25th and 75th percentiles).
Categorical data are summarized as numbers and percentages.
Abbreviations: BMI, body mass index; HbA1c, hemoglobin A1c; IQR, interquartile range.
[1]Data collected from September 2022 to August 2023.
[2]Diabetes-related complications include neuropathy, retinopathy, nephropathy, cardiovascular disease.
[3]All types of appointments include dietitians, endocrinologists, diabetes or ophthalmic nurses, and podiatrists.
